# Concise Reviews: Assisted Reproductive Technologies to Prevent Transmission of Mitochondrial DNA Disease

**DOI:** 10.1002/stem.1887

**Published:** 2015-02-17

**Authors:** Jessica Richardson, Laura Irving, Louise A Hyslop, Meenakshi Choudhary, Alison Murdoch, Douglass M Turnbull, Mary Herbert

**Affiliations:** a Wellcome Trust Centre for Mitochondrial Research, Newcastle UniversityNewcastle upon Tyne, United Kingdom; b Institute of Genetic Medicine, Newcastle UniversityNewcastle upon Tyne, United Kingdom; c Institute of Neuroscience, Newcastle UniversityNewcastle upon Tyne, United Kingdom; d Newcastle Fertility Centre, Newcastle Hospitals NHS Foundation TrustNewcastle upon Tyne, United Kingdom

**Keywords:** Zygote, Mitochondria, Mitochondrial DNA, Preimplantation genetic diagnosis, Pronuclear transfer, Spindle transfer

## Abstract

While the fertilized egg inherits its nuclear DNA from both parents, the mitochondrial DNA is strictly maternally inherited. Cells contain multiple copies of mtDNA, each of which encodes 37 genes, which are essential for energy production by oxidative phosphorylation. Mutations can be present in all, or only in some copies of mtDNA. If present above a certain threshold, pathogenic mtDNA mutations can cause a range of debilitating and fatal diseases. Here, we provide an update of currently available options and new techniques under development to reduce the risk of transmitting mtDNA disease from mother to child. Preimplantation genetic diagnosis (PGD), a commonly used technique to detect mutations in nuclear DNA, is currently being offered to determine the mutation load of embryos produced by women who carry mtDNA mutations. The available evidence indicates that cells removed from an eight-cell embryo are predictive of the mutation load in the entire embryo, indicating that PGD provides an effective risk reduction strategy for women who produce embryos with low mutation loads. For those who do not, research is now focused on meiotic nuclear transplantation techniques to uncouple the inheritance of nuclear and mtDNA. These approaches include transplantation of any one of the products or female meiosis (meiosis II spindle, or either of the polar bodies) between oocytes, or the transplantation of pronuclei between fertilized eggs. In all cases, the transferred genetic material arises from a normal meiosis and should therefore, not be confused with cloning. The scientific progress and associated regulatory issues are discussed. Stem Cells *2015;33:639–645*

## Introduction

The fertilized human egg contains two types of DNA: the nuclear DNA, which is packaged into chromosomes, is inherited from both parents and enclosed in two haploid pronuclei (PN). By contrast, the DNA within mitochondria (mtDNA) consists of small (16.5 kb) circular molecules, which are packaged into nucleoprotein complexes, called nucleoids, and inherited exclusively through the female lineage 1. The small number of mitochondria introduced by the sperm is targeted for destruction by a conserved autophagic mechanism known as mitophagy 2. Thus, the mtDNA (>100,000 copies) present in the human oocyte 3 constitutes the founder population for the mitochondria in all the cell types of the resulting embryo.

According to the endosymbiotic theory, mtDNA is the remnant of the genome of a once free-living α-proteobacterium, which has been engulfed by the ancestor of the modern-day eukaryotic cell 4,5. As a result, the outer membrane of mitochondria is related to the eukaryotic plasma membrane while the inner membrane has retained some prokaryotic properties. While the main function of mitochondria is to produce ATP by oxidative phosphorylation (OXPHOS), they have acquired additional functions during evolution including induction of apoptosis 6,7, calcium homeostasis 8, and the formation of iron sulfur clusters 9. Although mitochondria contain their own DNA, their biological functions are dependent upon nuclear-encoded genes, whose protein products are imported across the mitochondrial membranes 10. Of the estimated ∼1,100 genes required for mitochondrial function in humans, only 37 are encoded by the mtDNA. These include 13 polypeptides, together with two rRNAs and 22 tRNAs required for mitochondrial protein synthesis 11. All proteins encoded by mtDNA are components of the OXPHOS system, which consists of five large protein complexes containing a total of 80–100 subunits 11. Thus, the production of ATP requires direct interaction between proteins encoded by the mitochondrial and the nuclear genome.

## Inheritance of Mitochondrial DNA Disease

Since mitochondrial genes are essential for the OXPHOS system, mutations in mtDNA can reduce mitochondrial ATP production, which particularly affects organs with high energy requirements, such as the brain, muscle, and heart 12,13. Mutations can be present in all copies of mtDNA (homoplasmy), or in only a fraction of copies (heteroplasmy), and the severity of clinical symptoms is determined by the ratio of mutated to wild-type mtDNA 14. Diseases associated with mtDNA mutations include a broad range of debilitating and fatal conditions, none of which can be currently cured 12,13. Whereas the estimated incidence of mtDNA disease in adults is 1 in 5,000 15, low levels of pathogenic mutations are more prevalent and have been detected in 1 out of every 200 births 16,17.

Mutated copies of mtDNA present in the oocyte are transmitted to the embryo. In the case of women with homoplasmic mtDNA mutations, the entire complement of mtDNA carries the mutation therefore all oocytes are affected. By contrast, the oocytes of women with heteroplasmic mtDNA contain variable mtDNA mutation loads. This is thought to be due to a phenomenon known as the mtDNA genetic bottleneck, which involves a dramatic decline in mtDNA copy number during female germ cell development, giving rise to a statistical sampling effect which results in marked variation in the level of heteroplasmy between individual oocytes 11,18,19. As a consequence, the transmission of mtDNA disease from a woman with a heteroplasmic mtDNA mutation to her children is unpredictable. Given the difficult reproductive choices faced by women carrying pathogenic mtDNA mutations, there is a growing interest in the development of assisted reproductive technologies to prevent transmission of mtDNA mutations from mother to child.

## Preimplantation Genetic Diagnosis for Detecting mtDNA Mutations

Preimplantation genetic diagnosis (PGD) is an established procedure for preventing transmission of mutations in nuclear DNA. Embryos are tested by removing one or more cells for genetic analysis. Unaffected embryos are then chosen for transfer to the uterus. In recent years, PGD has been applied to reduce the risk of transmitting mtDNA disease. However, clinical decisions related to the transfer of embryos following PGD for mtDNA mutations are complicated by the need to define thresholds of heteroplasmy, which can vary between mutations 14. The question of whether the sampled cells are representative of the entire embryo is also fundamental to the success of PGD for reducing the risk of mtDNA disease.

A number of strategies could be used to predict the mtDNA mutation load in embryos. For example, it has been proposed that polar bodies, which are the by-products of female meiosis, provide a reliable and minimally invasive proxy for the oocyte mutation load 20,21. However, other studies in mouse 22 as well as humans 23,24 indicate a low correlation in mtDNA mutation load between the polar bodies and the oocyte. This may be linked to the highly asymmetric segregation of mitochondria during female meiosis 25. An alternative and commonly used approach is to remove cells from the developing embryo, which is typically performed at the eight-cell stage. Data from human embryos indicate a low variability in the level of heteroplasmy between the blastomeres of cleavage-stage embryos [26–30]. It has also been reported that trophectoderm cells biopsied from human blastocysts are representative of the mtDNA mutation load in the inner cell mass (ICM) 26. However, there are conflicting reports on the mutation load of a child born following trophectoderm biopsy 26,31, which may be linked to the assays used to measure mtDNA.

Experimental systems to study the segregation of variant mtDNA during early development include either embryos produced by females with heteroplasmic mtDNA mutations or those in which heteroplasmy has been induced artificially. However, an elegant set of experiments performed by Meirelles and Smith indicated that variant mtDNA segregates more uniformly between blastomeres when it is inherited through the germ line rather than introduced via karyoplast or cytoplast fusion with a fertilized egg 32. Interestingly, the widest variation was observed following cytoplast fusion 32. Consistent with this, it has recently been reported that fusion of bisected oocytes from rhesus macaque females with distinct mtDNA haplotypes resulted in embryos with widely varying levels of heteroplasmy between cells 33. It seems likely that in this experimental system, the orientation of the plane of the first embryonic cleavage relative to the plane of oocyte fusion is crucial for determining the fate of variant mtDNA. Indeed, it has been suggested previously that experimental models involving artificially induced heteroplasmy may be of limited relevance to our understanding of how inherited mtDNA mutations segregate during early human development 29.

In conclusion, current evidence suggests that there are no major shifts in the segregation of inherited mtDNA mutations during early embryonic development. Thus, blastomeres removed from eight-cell embryos are likely to provide a reliable indication of the mutation load in the entire embryo. Therefore, PGD constitutes a promising risk reduction strategy for affected families.

## Uncoupling the Inheritance of Nuclear and mtDNA to Prevent Transmission of mtDNA Disease

While PGD can be used to identify embryos with low mutation loads, it is not effective for women with homoplasmic mtDNA mutations or women with heteroplasmic mtDNA mutation loads close to the disease threshold. To address this issue, recent research has focused on the development of techniques to reduce the risk of transmission of mtDNA disease by transplanting the nuclear genome between oocytes before or after fertilization with the aim of uncoupling the inheritance of nuclear and mtDNA. In principle, this would enable women who carry mtDNA mutations to have a genetically related child while greatly reducing the risk of transmitting mtDNA mutations.

**Figure 1:**
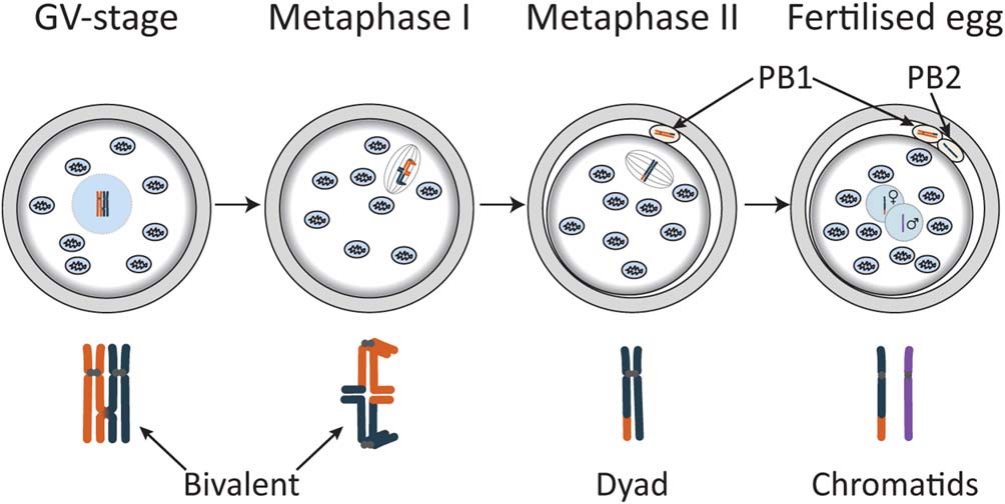
Schematic drawing showing progression from prophase of meiosis I (GV stage) to completion of meiosis II following fertilization. The diploid maternal genome contained in the large nucleus (GV) of the prophase I oocyte is packaged into bivalent chromosomes formed during meiotic recombination when pairs of replicated parental homologs become linked at the sites of reciprocal DNA exchange. Oocytes enter M phase of first meiotic division (MI) in response to hormonal stimulation and undergo anaphase of MI when bivalents are converted to dyad chromosomes, consisting of a pair of chromatids (at least one of which is a recombinant). Half of the dyads are ejected in the first polar body. The dyads remaining in the oocyte align on the second meiotic division (MII) spindle poised to undergo anaphase in response to sperm entry. During anaphase of MII dyads are resolved to single chromatids and half is lost in the second polar body. The chromatids remaining in the oocyte become surrounded by a nuclear membrane to form the female pronucleus. The products of the first and second meiotic divisions each contain a unique genome. Abbreviation: GV, germinal vesicle.

Transplantation of the nuclear genome can be either performed on the fertilized egg, or before the oocyte is fertilized, for which the process of female meiosis offers several options (Fig. 1). Throughout its growth phase, the oocyte remains arrested in prophase of meiosis I with a very large nucleus known as the germinal vesicle (GV). The GV contains bivalent chromosomes formed during meiotic recombination when replicated maternal and paternal homologs become physically linked at sites of reciprocal exchange of DNA between nonsister chromatids to form crossovers, which in cytological studies are known as chiasmata 34. In the sexually mature female, a hormonal stimulus induces the fully grown oocytes to enter M phase of meiosis I. During the first meiotic division, crossovers are resolved and half of the resulting dyad chromosomes (each containing two chromatids) are expelled into the first polar body. This occurs shortly before the oocyte is ovulated. The dyad chromosomes remaining in the oocyte align on the meiosis II spindle and the oocyte remains arrested at this stage until sperm entry. This triggers the second meiotic division (MII) during which one chromatid of each chromosome is retained in the oocyte while the other is expelled into the second polar body. The haploid genomes from the oocyte and the sperm are then separately packaged into pronuclei (Fig. 1). Thus, in contrast to spermatogenesis, which produces four equal-sized gametes, female meiosis produces only one gamete capable of fertilization, the oocyte, which retains a haploid set of chromosomes and most of the cytoplasm.

**Figure 2:**
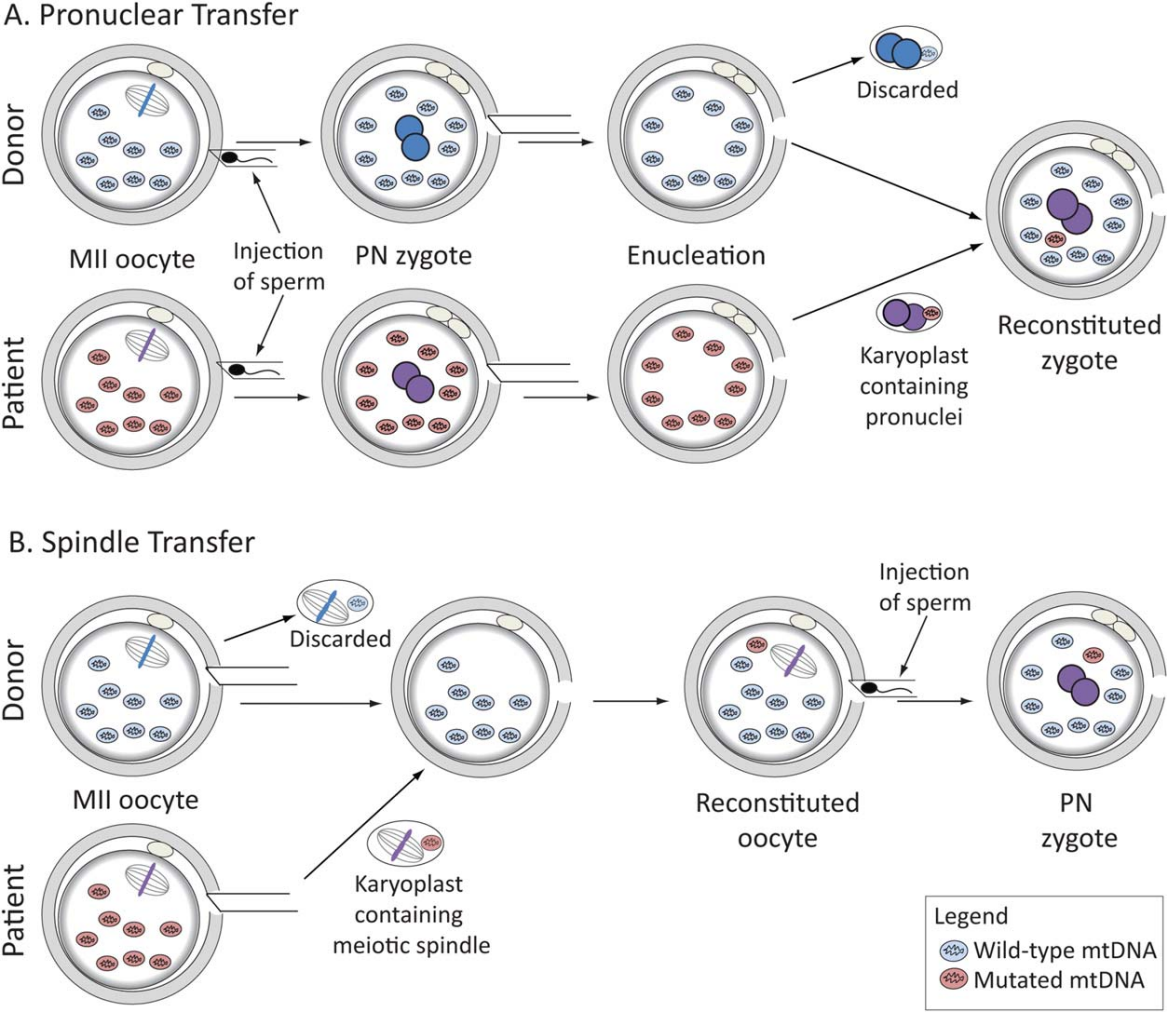
Schematic drawing showing approaches to meiotic genome transfer that has been tested in human oocytes/zygotes for potential clinical application to reduce the risk of transmitting mtDNA disease. (A): Pronuclear transfer: MII-arrested oocytes obtained from the affected woman and a healthy donor are fertilized and the pronuclei are transferred in a karyoplast from the affected woman's fertilized egg to the enucleated donor egg. (B): Spindle transfer: oocytes obtained from an affected woman and a healthy donor are enucleated by removal of the MII spindle and its chromosomes in a karyoplast. The karyoplast from the affected woman is fused with the enucleated oocyte from the healthy donor. Reconstituted oocytes are then fertilized and undergo the second meiotic division followed by formation of the male and female pronuclei. Abbreviations: MII, second meiotic division; PN, pronuclei.

The technique of pronuclear transfer (PNT) between fertilized mouse eggs (zygotes) was pioneered by McGrath and Solter more than 3 decades ago 35. The procedure involves treatment of zygotes with microtubule and actin-depolymerizing drugs to facilitate removal of the pronuclei without the need to penetrate the plasma membrane. The PN are pinched-off within a small volume of membrane-enclosed cytoplasm, known as a karyoplast, which is subsequently fused with an enucleated zygote. Membrane fusion is facilitated by inactivated Sendai virus or an electrical pulse 35,36. However, the latter is not well tolerated by human oocytes and zygotes 37,38. The experiments of McGrath and Solter 35 revealed that PNT between zygotes from different mouse strains can produce healthy and normally reproducing offspring. The possibility of using PNT to prevent transmission of mtDNA disease (Fig. 2A) was first proposed in the 1990s 21. Later experiments using zygotes from a mouse carrying a rearrangement in the mtDNA indicated that the fraction of mutant mtDNA could be greatly reduced by PN transfer into enucleated zygotes from females with wild-type mtDNA 21. More recently, proof of concept experiments with abnormally fertilized eggs explored the potential of PNT in human zygotes 38. Despite their large size (25–30 µm), transplantation of PN between human zygotes was technically feasible and compatible with development to the blastocyst stage 38. After optimization of the procedure, the level of the mtDNA “carried-over” within the karyoplast was reduced to <2% on average 38, which is well below the disease threshold for mutations studied to date 14.

In relation to transplantation of the nuclear genome before the oocyte is fertilized, it is, in theory, possible to harvest immature oocytes and to transplant the GV (Fig. 1). However, this would require in vitro maturation of oocytes from the GV stage to the MII stage, which is the stage at which oocytes are conventionally harvested for IVF treatment. However, GV transfer would necessitate removal of the cumulus cells, which are thought to be important for normal maturation of the oocyte 39,40. It is therefore likely that successful adoption of this approach would require strategies that compensate for the absence of an intact cumulus-oocyte complex. So far, there are no reports of successful GV transfer between human oocytes.

Currently, the most promising strategy for human oocytes is to transfer the nuclear genome between MII-arrested oocytes (Fig. 2B). In contrast to the GV or the PN stage, the MII oocyte chromosomes are not enclosed within a nuclear membrane. Instead, they are aligned on the MII spindle, poised to undergo anaphase II following sperm entry. As conventional light microscopy cannot visualize the spindle or chromosomes, the standard practice is to use liquid crystal birefringence, which enables spindle visualization [37, 41–43]. However, this approach may be problematic in cases where chromosomes become misaligned or scattered, as has been reported for oocytes from older women 44. The use of a fluorescent DNA dye such as Hoechst is not desirable, because they intercalate into DNA and require damaging UV light for excitation.

Despite these technical challenges, proof-of-concept experiments with rhesus macaque oocytes resulted in the birth of healthy monkeys with undetectable mtDNA carryover 45. Follow-up experiments using human oocytes indicated that MII spindle transfer (MST) results in a low level of mtDNA carryover 41,43. However, fertilization of MST oocytes resulted in a high incidence (48%) of zygotes containing an abnormal number of pronuclei 41. This was, at least in part, due to premature chromatid separation in the absence of second polar body formation, causing both sets of maternal chromatids to remain in the oocyte. Interestingly, abnormal numbers of pronuclei were not observed upon fertilization of MST oocytes from monkey 45, indicating that human oocytes are more sensitive to premature chromatid separation. Nevertheless, a high proportion of those that underwent normal fertilization developed to the blastocyst stage 41. However, no data were presented on blastocyst morphology, which correlates closely with implantation potential 46,47.

Embryonic stem cells (ESCs) derived from human blastocysts produced following MST showed normal expression of pluripotency markers 41,43, normal metabolic profiles 43, and those that were derived from fertilized embryos showed a normal karyotype 41. Analysis of mtDNA revealed that very low levels, typically <1%, of karyoplast-associated mtDNA persisted in ESC lines and their derivatives 41,43. Together, these data indicate that the epiblast precursor cells from human MST blastocysts behave normally following explantation of the ICM. However, the establishment of viable pregnancies also requires that the blastocyst's trophectoderm and primitive endoderm lineages are competent to develop into the placenta and yolk sac, respectively. Therefore, it will be important to investigate in more detail blastocyst morphology, lineage specification, and gene expression of blastocysts following MST and PNT.

More recently, experiments in mice indicated that the oocyte polar bodies could be used as a source of nuclear DNA with minimal carryover of mtDNA 48. Fusion of first polar bodies with enucleated, unfertilized oocytes resulted in efficient blastocyst formation following fertilization and six pups were born 48. Second polar bodies, which, like the pronucleus, contain one chromatid from each chromosome, were fused with zygotes from which the female pronucleus had previously been removed. Blastocysts and live births were obtained, but less efficiently compared with the first polar body/oocyte fusions 48. If found to be effective in humans, the use of polar bodies as a source of additional haploid maternal genomes has the potential to reduce the number of oocytes required from women affected by mtDNA mutations.

In conclusion, transplantation of the nuclear genome can be performed at various stages of female meiosis and the haploid genomes generated following fertilization contain a unique mix of genes, reshuffled during meiotic recombination and stochastically inherited during the subsequent meiotic divisions. Thus, to avoid any possible confusion with the process of cloning, it is important to stress that all products of meiosis (pronuclei and polar bodies) are genetically unique. Given the range of possible approaches to transplanting the nuclear genomes of the oocyte or zygote, an umbrella term such as meiotic nuclear transfer Mei-NT might best describe the general approach, while making it clearly distinct from cloning.

## Toward Clinical Treatment

In many countries, the introduction of these novel Mei-NT techniques as clinical treatments to prevent transmission of mtDNA disease will involve changes to existing regulations governing assisted conception treatments. In the U.K., amendments to the Human Fertilization and Embryology Act in 2008 included provision for the law to be changed by Parliament to enable the Human Fertilization and Embryology Authority (HFEA) to licence the use of new techniques for the purpose of preventing transmission of mtDNA disease. The ethical issues associated with the introduction of these techniques have been comprehensively reviewed elsewhere 49. Following a number of broadly supportive public consultations, the proposed changes to current legislation have been drafted. The next step will be a debate followed by a vote in both Houses of Parliament. In the event that Parliament approves the changes, IVF centers would be required to apply to the HFEA for a licence to offer the techniques in clinical treatment. This would be subject to additional corroborative evidence on the robustness and efficiency of PNT/MST as specified by a panel of experts convened by the HFEA in the U.K. 50.

In the U.S., the Food and Drug Administration recently held a public horizon-scanning meeting to explore whether it would be appropriate to approve clinical trials to test the safety and efficacy of MST/PNT. In contrast to the U.K., the use of these techniques might not necessarily be confined to reducing the risk of mtDNA disease. It has been proposed that transfer of the MII spindle from the oocyte of an older woman to the enucleated oocyte of a younger woman might be effective in ameliorating the effects of female age on fertility 51. However, it is been well established that the majority of MII human oocytes from older women are either already aneuploid 52, or contain pairs of single chromatids 53,54, which have a high risk of missegregating during MII. Currently, there is no known mechanism whereby these chromosomal aberrations could be reversed by transferring the MII spindle to the enucleated oocyte obtained from a younger woman.

Given the low levels of mtDNA carryover during PNT 38 and MST 41,43 in humans, and polar body transfer in mice 48, these techniques are likely to be highly effective in reducing the risk of mtDNA disease in children of affected women. However, it has been proposed that coevolution of the mitochondrial and maternal nuclear genomes might result in adverse effects arising from the creation of a new combination of mtDNA and nuclear DNA. Such concerns are based on evidence of adverse outcomes following experimentally induced heteroplasmy in mice and *Drosophila* 55. However, there is no evidence for incompatibilities between nuclear and mitochondrial genotypes in humans, even in couples with divergent mitochondrial haplotypes 56. Thus, the theoretical risk associated with incompatibilities of nuclear and mitochondrial genomes seems remote in comparison to the very real risk of serious disease in children of women who carry high levels of pathogenic mtDNA mutations.

Ongoing in vitro experiments to optimize the techniques and to test the effects of Mei-NT on preimplantation development will require a supply of donated human oocytes. Furthermore, a continued supply will be required in the event of the techniques being translated to clinical treatment. Practices related to oocyte donation vary widely 57. Commonly used options include so called “egg sharing,” in which women undergoing IVF treatment donate half of their oocytes for use by others. Such schemes are already well established as a source of donated oocytes for IVF treatment 58,59. Alternatively, oocytes can be obtained from women who donate altruistically 60,61.

In conclusion, while PGD can reduce the risk of transmitting mtDNA disease, it relies on the production of embryos with low levels of mtDNA mutation. By contrast, Mei-NT techniques to replace mutated mtDNA with wild-type mtDNA offer women with high mutation loads the possibility to have a genetically related child without the risk of transmitting disease.
